# Draft Genome Sequence of Bacteriophage vB_Eco_swan01

**DOI:** 10.1128/genomeA.00501-17

**Published:** 2017-07-13

**Authors:** Slawomir Michniewski, Tamsin Redgwell, David J. Scanlan, Andrew D. Millard

**Affiliations:** a School of Life Sciences, University of Warwick, Coventry, United Kingdom; b Microbiology and Infection Unit, Warwick Medical School, University of Warwick, Coventry, United Kingdom

## Abstract

Bacteriophage vB_Eco_swan01 was isolated from an ornamental pool using *Escherichia coli* MG1655 as the host. Bacteriophage vB_Eco_swan01 has limited similarity with other known phages at the nucleotide level and likely represents a new bacteriophage species within the *Tunavirinae*.

## GENOME ANNOUNCEMENT

Here, we report the genome sequence of bacteriophage vB_Eco_swan01, which is capable of infecting *Escherichia coli* MG1655. This phage was isolated using an enrichment procedure involving a single-agar-layer plaque assay (1). Briefly, an 80-mL filtered water sample (i.e., filtered through 0.45-µm pores) was mixed with 5 mL of *E. coli* MG1655 cells, CaCl_2_ to a final concentration of 1 mM, and incubated at room temperature for 10 min. Subsequently, 80 mL of molten 1.2% (wt/vol) LB agar (previously adjusted to 50°C) was added to the sample. The mixture was then poured into disposable petri dishes and incubated overnight at 37°C. A single plaque of ~3 mm in diameter was picked and transferred into SM buffer, followed by vortexing to release phages from the agar plug and storage at 4°C. Bacteriophage genomic DNA was extracted from a lysed culture using a phenol:chloroform method (2). Genomic DNA was prepared using the NexteraXT DNA sample preparation kit (Illumina) with modified PCR conditions consisting of 14 rounds of 97°C for 10 s, 55°C for 30 s, and 65°C for 1 min. Sequencing was performed on the MiSeq platform using V2 (2 × 250-bp) chemistry. The resulting FASTQ files were trimmed with Sickle using default parameters (3), prior to being assembled with SPAdes version 3.7 using the “-careful” option (4). The genome was sequenced to an average depth of 344×. The resulting single contig was annotated with Prokka version 1.11 using a custom database constructed from all complete viral genomes within the European Nucleotide Archive (5) and then further annotated using hmmscan using the prokaryotic Viral Orthologous Groups (pVOG) collection of hmm profiles (6), using a cutoff value of <1E−15. Bacteriophage vB_Eco_swan01 has a double-stranded DNA genome of 50.865 kb and a G+C content of 44.96%. A total of 83 open reading frames were predicted, with no tRNAs found. At the nucleotide level, vB_Eco_swan01 has an average nucleotide identity (ANI) of 72% with bacteriophage pSF1, an unclassified member of the family *Siphoviridae*, but has no significant similarity with other bacteriophages. For the majority of genes, it was not possible to predict a function for the encoded proteins. Of the 83 predicted proteins, 75 could be annotated as being part of a known pVOG. Phylogenetic analysis using genes encoding for the tape measure protein (00017) and large terminase subunit (00031) revealed that vB_Eco_swan01 is related to phages in the subfamily *Tunavirinae*. However, given the low ANI compared to all other phages, vB_Eco_swan01 likely represents a new species based on current definitions of phage classification (7). This genome adds further diversity to those bacteriophages capable of infecting *E. coli*.

### Accession number(s).

The draft genome sequence of bacteriophage vB_Eco_swan01 has been deposited in DDBJ/ENA/GenBank under the accession number LT841304.
